# Pericardial injury with cardiac tamponade and bleeding from the pericardium confirmed using contrast-enhanced computed tomography: a case report

**DOI:** 10.1186/s40792-019-0584-y

**Published:** 2019-02-19

**Authors:** Jumpei Takamatsu

**Affiliations:** Department of Emergency Medicine, Kansai Rosai Hospital, Amagasaki, Japan

**Keywords:** Contrast-enhanced CT, Pericardial effusion, Hematoma, Bleeding

## Abstract

**Background:**

Simple pericardial injuries are asymptomatic in many cases and usually do not cause bleeding that leads to cardiac tamponade. In this study, however, we report a case involving a patient with pericardial injury, in whom extravasation in the pericardium was identified using contrast-enhanced computed tomography (CT).

**Case presentation:**

A 67-year-old man fell from a 3-m-high ladder and was injured and transported to our hospital. No pericardial effusion was observed on focused assessment with sonography for trauma (FAST) or plain CT on arrival, but pericardial effusion was detected on follow-up observation. Thereafter, his circulatory dynamics began to deteriorate. We then performed FAST to identify the bleeding source, but it was difficult to visualize on echocardiography. Thus, contrast-enhanced CT (CECT) was performed and extravasation was confirmed in the pericardium. We believed that the accumulation of pericardial effusion caused cardiac tamponade; hence, we performed emergent thoracotomy. When we released the cardiac tamponade, his circulatory dynamics improved, and we could stabilize the patient’s condition by ligating the bleeding vessel from the pericardium.

**Conclusion:**

If visualization is difficult on FAST, like in this case, CECT is useful for identifying the cause of pericardial effusion if circulatory dynamics can be determined. We were able to confirm that extravasation occurred from the pericardium using CECT; hence, we could confirm that pericardial injury caused bleeding and may cause cardiac tamponade. Thus, if cardiac tamponade is suspected, not only damage to the heart itself, but also damage caused by pericardial vascular injury should be considered. Further, if circulatory dynamics are stable, CECT should be performed.

## Background

In 1958, Parmley reported on pericardial injury based on the results of autopsy cases from the Armed Forces Institute of Pathology (AFIP) [1]. Blunt pericardial injury is said to occur from direct, high-energy impact or from transmitted sudden and acute increases in intraabdominal pressure. Most pericardial injuries, whether single or multiple, can occur after blunt injury, and many are associated with extensive cardiac injuries. However, they may also occur as isolated injuries. Simple pericardial injury is asymptomatic in many cases [2] and does not cause cardiac tamponade. Therefore, Parmley also reported that if pericardial laceration occurs as an isolated injury, it is usually of no consequence unless complicated by hemorrhage from a lacerated pericardiophrenic artery [1]. That is, pericardial injury accompanied by pericardiophrenic arterial laceration can result in fatal injury, and thus, detection of a pericardiophrenic arterial laceration has great diagnostic significance. However, there have been no reports that contrast-enhanced CT (CECT) confirmed bleeding from the pericardium.

CECT may be useful for confirming the bleeding point if thoracic injury is suspected. Unless there is an obvious obstruction of the pericardium or the heart is in an abnormal position, pericardial injury without cardiac injury is not usually identifiable by preoperative imaging studies. In this case, we confirmed that extravasation occurred from the pericardium using CECT. Pericardial effusion accumulated as a result of the bleeding and based on this finding, it was thought that cardiac tamponade occurred due to damage of the pericardium without cardiac injury. We encountered a case wherein we performed emergency thoracotomy and confirmed the pericardial injury without cardiac injury diagnosed with CECT. Pericardial injury occurring along with cardiac tamponade and bleeding from the pericardium was confirmed by CECT during the operation. Thus, CECT was found to be useful for diagnosing pericardial injury.

## Case presentation

**Fig. 1:**
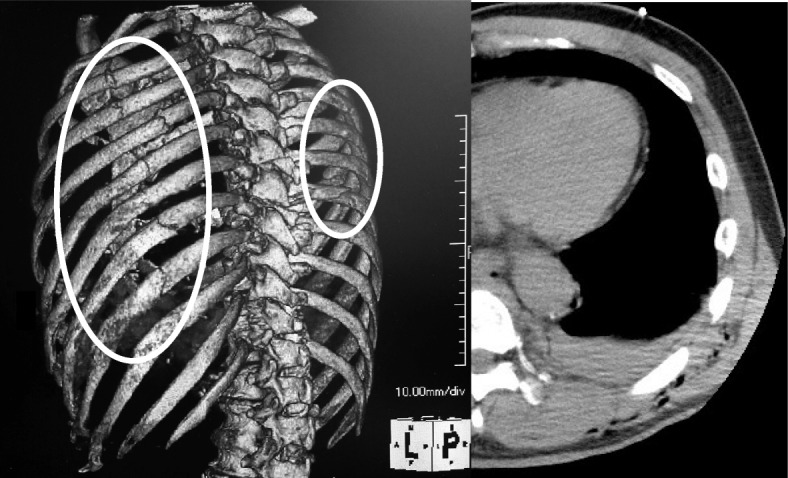
Plain computed tomography (CT) findings on admission. The left 3D-CT showed bilateral multiple rib fractures (circle), and there was no sign of pericardial injury on the right CT

A 67-year-old man, who had no significant family history and past history, accidentally fell from a stepladder, which was 3 m in height, while he was pruning a plant. Owing to the resulting injuries, he was transported to our hospital. After the fall, his consciousness level was Glasgow coma scale (GCS) E3V5M6, respiratory rate (RR) was 24 breaths per min, SpO 2 was 90% (oxygen 10 L/min reservoir mask), heart rate (HR) was 96 beats per min, and blood pressure was (BP) 173/103 mmHg. On arrival, the airway was opened, RR was 28/min, SpO 2 was 90% (oxygen 5 L/min mask), BP was 148/100 mmHg, HR was 104/min, body temperature was 36.0 °C, and focused assessment with sonography for trauma (FAST) was negative. Electrocardiography revealed sinus tachycardia and heart expansion was not observed in chest X-ray images. There was no jugular venous distention, and there were no heart noises on auscultation. There was no obvious bruise on the skin surface, but he was complaining of occipital pain and left back pain. Bilateral multiple rib fracture, left lung contusion, left hemothorax, and right pneumothorax were observed on plain whole-body CT (Fig. 1), but no pericardial effusion was observed. After CT, we performed chest drainage on both sides. Drainage after indwelling was barely observed on the right side, and drainage from the left side was 300 ml. After placing the thoracic drain, he was transferred to the ICU.

**Fig. 2:**
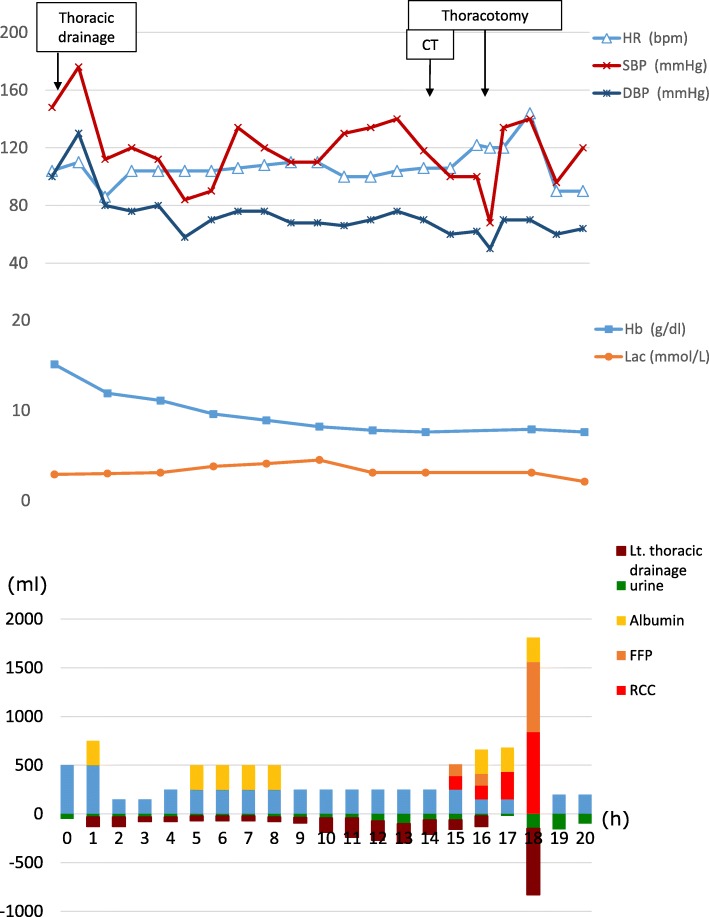
Clinical course after admission

**Fig. 3:**
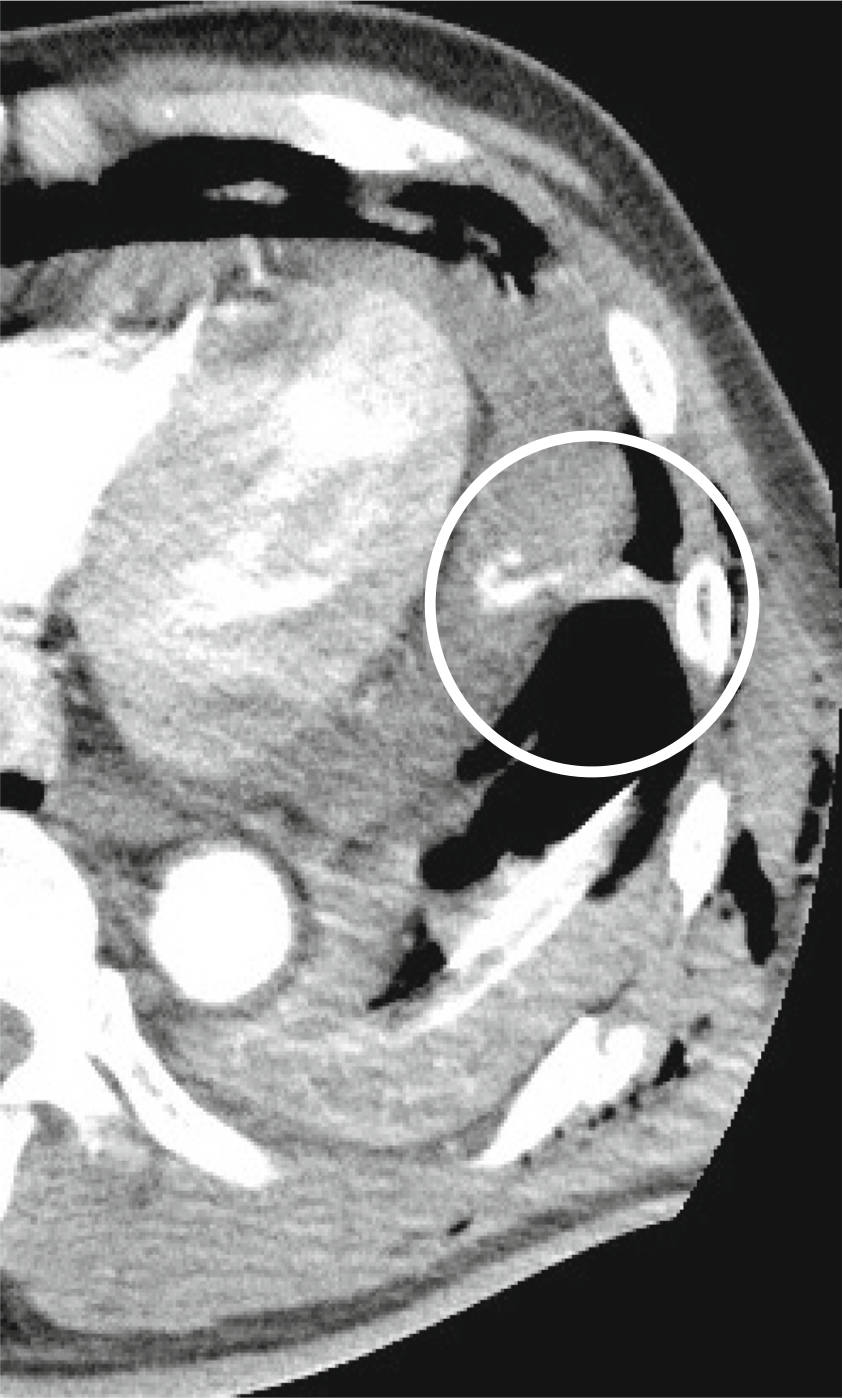
Dynamic CT scan finding at 10 h after admission. Dynamic CT scan showed active bleeding in the pericardium (circles)

The following was the clinical course after the hospitalization (Fig. 2): drainage from the left thoracic tube increased and reached almost 800 ml in 4 h from 10 h after admission. It was difficult to visualize his pericardial effusion and pleural effusion using echocardiography. As his circulatory dynamics were intact, CECT (Fig. 3) was performed to investigate the cause of the massive hemothorax. Subcutaneous emphysema was found in the chest wall. Furthermore, we found a “flattened heart sign,” suggesting pericardial effusion with extravasation and cardiac tamponade in the pericardium. After returning to the ICU, we were preparing for surgery to stop the bleeding. His HR rapidly deteriorated to 120 beats/min and BP was also 68/50 mmHg. Therefore, we determined that cardiopulmonary arrest due to cardiac tamponade was imminent. After tracheal intubation, we performed left anterior lateral thoracotomy. We identified slight bleeding that included the pulmonary parenchyma after aspiration of blood stored in the thoracic cavity. We stopped the bleeding from his chest wall by applying a thoracotomy device; hence, we thought that his massive hemothorax was caused by bilateral multiple rib fractures. We removed a hematoma that formed in the pericardial fat. Furthermore, there was no obvious open wound on the pericardium. We confirmed that there was no phrenic nerve near the hematoma and we performed a pericardiotomy on the portion where a hematoma had formed. We incised the pericardium, which resulted in a blood spurt. We then removed the hematoma in the pericardium and sutured the ruptured pericardiophrenic artery including pericardial fat, thus achieving hemostasis. The source of the damage to the heart was unclear. After pericardiotomy, his HR did not change at 120 beats/min, but his BP improved to 134/70 mmHg. We performed temporary thoracic closure with negative-pressure wound therapy using the VAC® system (Acelity L.P. Inc., San Antonio, TX) as damage control management. The operation duration was 75 min. The total amount of blood was 530 ml in the thoracic cavity. After 12 h, when we removed the VAC® system and checked the pericardium, hemostasis was achieved. Closure of the pericardium was difficult because the pericardium did not stretch. Therefore, we did not attempt to close the pericardium and we closed the chest. The postoperative course was good. A ninth thoracic spinal burst fracture was associated with the fall; hence, the patient was forced to carry out long-term bed rest. On day 41, he was discharged wearing a corset.

## Conclusions

To diagnose cardiac and pericardial injury, FAST, plain X-ray imaging, or CT can be performed [3, 4]. The sensitivity of FAST to detect pericardial effusion is considered to be extremely high; [5] however, in chest trauma with subcutaneous emphysema, it is sometimes impossible to sufficiently visualize pericardial effusion [6]. Since this case also involved subcutaneous emphysema, we were unable to visualize pericardial effusion using FAST. Thus, in order to identify the cause of intrathoracic bleeding, pericardial injury with bleeding can be diagnosed using CECT while maintaining circulatory dynamics.

We confirmed extravasation from the pericardium using CECT. This indicates that bleeding in the pericardium may be mistaken as pericardial effusion even in cases of pericardial injury. Cardiac injury with pericardial effusion is either a slight patched pericardial laceration or the absence of pericardial laceration. A previous report presented images showing extravasation from cardiac injury [7, 8]. However, there is no report showing images of bleeding from the pericardium. In this case, although the circulation dynamics were unstable, we determined that it could withstand movement. By performing CECT, we were able to capture an image of bleeding from the pericardium.

If FAST cannot sufficiently help in visualizing the pericardium and if circulatory dynamics permit, CECT is useful for identifying the cause of pericardial effusion as observed in this case. If cardiac tamponade is suspected, CECT should be performed as cardiac tamponade may occur not only due to injury to the heart itself, but also due to damages to the pericardium following arterial injury.

In this case, CECT was not performed on arrival. However, if there is a possibility of high energy trauma for the trunk, CECT should be performed at this stage as pericardial effusion may not be evident upon presentation but can occur later. In conclusion, in chest injuries in which the circulatory dynamics become gradually unstable, the patient should undergo multiple CECT examinations especially when visualizing critical damage by other examinations is not possible.

## Abbreviations

BP: Blood pressure
CECT: Contrast-enhanced computed tomography
CT: Computed tomography
FAST: Focused assessment with sonography for trauma
GCS: Glasgow coma scale
HR: Heart rate
RR: Respiratory rate
